# Inkjet-Printed Electron Transport Layers for Perovskite Solar Cells

**DOI:** 10.3390/ma14247525

**Published:** 2021-12-08

**Authors:** Dongli Lu, Wei Zhang, Lars Kloo, Liubov Belova

**Affiliations:** 1 Department of Materials Science and Engineering, KTH Royal Institute of Technology, SE-10044 Stockholm, Sweden; donglil@kth.se; 2 Department of Chemistry, Applied Physical Chemistry, KTH Royal Institute of Technology, SE-10044 Stockholm, Sweden; wzha@kth.se (W.Z.); larsa@kth.se (L.K.)

**Keywords:** inkjet printing, electron transport layers, perovskite solar cells, TiO_2_, SrTiO_3_, SnO_2_, cosolvent system

## Abstract

Inkjet printing emerged as an alternative deposition method to spin coating in the field of perovskite solar cells (PSCs) with the potential of scalable, low-cost, and no-waste manufacturing. In this study, the materials TiO_2_, SrTiO_3_, and SnO_2_ were inkjet-printed as electron transport layers (ETLs), and the PSC performance based on these ETLs was optimized by adjusting the ink preparation methods and printing processes. For the mesoporous ETLs inkjet-printed from TiO_2_ and SrTiO_3_ nanoparticle inks, the selection of solvents for dispersing nanoparticles was found to be important and a cosolvent system is beneficial for the film formation. Meanwhile, to overcome the low current density and severe hysteresis in SrTiO_3_-based devices, mixed mesoporous SrTiO_3_/TiO_2_ ETLs were also investigated. In addition, inkjet-printed SnO_2_ thin films were fabricated by using a cosolvent system and the effect of the SnO_2_ ink concentrations on the device performance was investigated. In comparison with PSCs based on TiO_2_ and SrTiO_3_ ETLs, the SnO_2_-based devices offer an optimal power conversion efficiency (PCE) of 17.37% in combination with a low hysteresis. This work expands the range of suitable ETL materials for inkjet-printed PSCs and promotes the commercial applications of inkjet printing techniques in PSC manufacturing.

## 1. Introduction

Electron transport layers (ETLs), which effectively collect photo-generated electrons from the light-absorbing perovskite material and transport these electrons to the conductive contact layer, are critical for fabricating efficient perovskite solar cells (PSCs). TiO_2_ is the most widely used electron transport material in the community of PSCs because of its chemical robustness, abundance, low cost, and good chemical and thermal stability, as well as good conduction band alignment with the perovskite [1,2]. A bilayer ETL consisting of a compact TiO_2_ (c-TiO_2_) film and a mesoporous TiO_2_ (mp-TiO_2_) layer is preferred for highly efficient PSCs [3,4]. Such a morphology was shown to offer a power conversion efficiency (PCE) exceeding 25% [5,6,7]. However, concerns regarding performance degradation of TiO_2_-based devices under long-time UV illumination [8] together with hysteresis and recombination problems caused by inefficient charge transfer at the TiO_2_/perovskite interface [1,9] remain. Although much effort was devoted to overcoming these issues and to promoting the device performance to new levels by employing doping [10], graphene/TiO_2_ composites [11,12], surface passivation [13], and interface engineering [14,15], the phenomenon of scan-direction hysteresis when using TiO_2_ ETLs in PSCs is still difficult to suppress [16]. Investigation of alternative electron transport materials may be a more effective strategy than tedious optimization efforts involving TiO_2_.

SrTiO_3_ is one of those alternative electron transport materials. As compared to that of TiO_2_, SrTiO_3_ exhibits a similar band gap of 3.2 eV and, despite its slightly higher conduction band energy level, good band alignment with the perovskite material. This is beneficial for a high open-circuit voltage (*V_OC_*) of SrTiO_3_-based PSCs [17]. The high electron mobility (5–8 cm^2^/(V·s)) of the materials may promote charge transport and help to reduce recombination losses [18]. There were already some reports focused on the applications of SrTiO_3_ ETLs in PSCs. It was an early stage reported that devices employing commercial SrTiO_3_ nanoparticles (average diameter ≤ 100 nm)) exhibited higher *V_OC_* and lower short-circuit current density (*J_SC_*) than PSCs based on TiO_2_ because of the large particle size of the SrTiO_3_ nanoparticles [17]. Later, graphene/SrTiO_3_ composites [19] and TiO_2_/SrTiO_3_ composites [20,21] were applied as ETLs to compensate for the low current density and retain the advantage of the high open-circuit voltage. Afterwards, PSCs based on a compact SrTiO_3_ ETL with a smaller particle size showed an improved stability and enhanced electron transport in the SrTiO_3_ ETL [18]. With the help of doping [8] and mixtures with another ETL [22], both the photovoltaic performance and stability of SrTiO_3_-based PSCs were improved.

SnO_2_ is considered to represent a promising alternative electron transport material compared to TiO_2_ because of its favorable electrical and chemical properties [23,24,25]. Its high electron mobility (100–200 cm^2^/(V·s)) and deep conduction band facilitate electron extraction and transport [26]. SnO_2_ also exhibits a wide bandgap (3.8 eV) and extensive stability [27,28] under UV illumination. After SnO_2_ was directly used as the ETL in a planar PSC in 2015 [25], to further improve the photovoltaic performance and stability of SnO_2_-based PSCs, efforts were made to adjust the electrical properties and to passivate the ETL/perovskite interface, such as employing mesoporous SnO_2_ [9,29,30], doping [31,32,33,34,35], a gradient interlayer [16], titanium (IV) chloride (TiCl_4_) treatment [36], or graphene [37].

The inkjet technology offers a solution to the emerging demands of additive patterning of functional multilayers and device components. The technology offers mask-free, cost-effective, and direct patterning processes, which are easily scalable to meter format. Therefore, the inkjet printing method shows a large potential for the up-scaling manufacturing of perovskite solar cells associated with less material consumption and negligibly small waste, in contrast to the conventionally employed spin-coating method. Inkjet printing was applied to fabricate functional layers relevant to PSCs, such as carrier transport layers [38,39,40], electrodes [41], and perovskite absorbers [42,43,44,45,46]. In this work, we focus on the inkjet-printed ETLs for PSCs. TiO_2_ ETLs were intensively investigated but not for inkjet-printed ETLs used in PSCs. Huckaba et al. fabricated inkjet-printed mp-TiO_2_ for PSCs and the champion cell offered a PCE of 18.29% [38]. Recently, Buffiere et al. reported that PSCs with inkjet-printed c-TiO_2_ ETLs exhibited a PCE of 13.7% [39]. High-performance SnO_2_ ETLs are usually prepared by spin coating [47], atomic layer deposition [48], electron beam evaporation [49] or dual-fuel combustion [50] methods, while there are few reports [40] focused on inkjet-printed SnO_2_. For the application of inkjet-printed SnO_2_ in PSCs, there is as far as we know only one recent report by Rohnacher et al., in which their best device displayed an efficiency of 18.8% [40]. To the best of our knowledge, there is no publication on inkjet-printed SrTiO_3_ ETL based PSCs. As mentioned before, the intrinsic properties of TiO_2_, such as the low mobility and instability under UV illumination, limit the photovoltaic performance of TiO_2_-based PSCs. Therefore, further research on inkjet-printed ETLs is still necessary for improving PCEs of inkjet-printed PSCs, and thus to make them comparable with or even superior to the spin-coated alternatives, and eventually promote the commercialization of inkjet-printed PSCs. Investigations of inkjet-printed SrTiO_3_ and SnO_2_ could expand the selection range of inkjet-printed ETLs for PSCs and pave the way for fully inkjet-printed and efficient PSCs.

In this study, we present inkjet printing processes for different ETLs for the application in PSCs by optimizing the ink design, the film uniformity and the device performance of devices based on these printed functional layers. The importance of the solvents used for dispersing TiO_2_ and SrTiO_3_ nanoparticles is investigated, and cosolvent systems are found to contribute to uniform film formation of mesoporous TiO_2_ and SrTiO_3_ ETLs. SrTiO_3_-based PSCs show higher PCE than TiO_2_-based devices. However, they suffer from lower current density and severe scan-direction hysteresis. Subsequently, TiO_2_ nanoparticles are introduced into the mesoporous SrTiO_3_ (mp-SrTiO_3_) layer to solve these issues by optimizing the TiO_2_ nanoparticle concentration in the SrTiO_3_ ink. The cosolvent system is also applied to print SnO_2_ ETLs and the effects of the precursor ink concentration on the device performance of SnO_2_-based PSCs are studied. Finally, an optimal PCE of 17.37% is achieved.

## 2. Materials and Methods

### 2.1. Materials

All reagents and solvents were purchased and used as received without further purification. Titanium (IV) isopropoxide (Ti(OCH(CH_3_)_2_)_4_, 97%) and titanium (IV) oxide powder (TiO_2_, ≥99.5%, 21 nm) were purchased from Sigma–Aldrich (Darmstadt, Germany). Strontium titanate nanoparticles (SrTiO_3_, 99.95%, 100 nm) were obtained from US Research Nanomaterials (Houston, TX, USA). Tin (IV) oxide (15% in H_2_O colloidal dispersion) was purchased from Alfa Aesar (Kandel, Germany). Lead iodide (PbI_2_, 99.99%) and lead bromide (PbBr_2_, >98.0%) were purchased from TCI (Tokyo, Japan). Formamidinium iodide (FAI, CH(NH_2_)_2_I, >98%) and methylammonium bromide (MABr, CH_3_NH_3_Br, >98%) were obtained commercially. Spiro-OMeTAD (99.8%) was purchased from Borun New Material Technology (Ningbo, China). Bis(trifluoromethane)sulfonimide lithium salt (LiTFSI, 99.95%), FK209 (Co(III) TFSI salt, 98%) and 4-*tert*-butylpyridine (TBP, 98%) were purchased from Sigma–Aldrich (Darmstadt, Germany).

### 2.2. Set-Up of Inkjet Printer

An inkjet station was constructed and used as a drop-on-demand inkjet printer in our lab. This system was designed for printheads from XaarJet to achieve flexible inkjet printing. In this work, XJ126/50 (126 active nozzles with a drop volume of 50 pL) printheads were used for printing TiO_2_ nanoparticle inks and XJ126/80 (126 active nozzles with a drop volume of 80 pL) printheads for other inks. More details about the technical information of the two printheads are shown in Table S1. The ejection voltage for all inks was set to 20 V and the printing frequency to 283.46 Hz. In this work, the inkjet printing of electron transport layers was operated under ambient conditions.

### 2.3. Device Fabrication

Before cleaning, fluorine-doped tin oxide (FTO) glass substrates (14 Ω/sq, Pilkington TEC) were cut into pieces with the size of 25 × 15 mm. One edge of each piece was etched with Zn powder and 2M HCl aqueous solution. Subsequently, these substrates were successively sonicated in a detergent solution (5% deconex in water), deionized water, acetone, and 2-propanol for 15 min. The clean substrates were stored in 2-propanol before use.

Before inkjet printing, the substrates were placed on a preheated printing stage at 60 °C. A compact TiO_2_ layer was inkjet-printed from a 0.125 M solution of titanium isopropoxide dissolved in 2-isopropoxyethanol, and then annealed at 450 °C for 45 min. On the top of the compact TiO_2_ layer, the mesoporous TiO_2_ layer was also inkjet-printed with an ink made by dispersing TiO_2_ nanoparticles in a mixture of ethanol and ethylene glycol (9/1, *v*/*v*). After printing, the substrate was annealed at 500 °C for 45 min. For SrTiO_3_-based devices, the compact TiO_2_ layer was fabricated using the same procedure as for the TiO_2_ based devices. The mesoporous SrTiO_3_ layer was inkjet-printed with a suspension ink of SrTiO_3_ nanoparticles dispersed in a mixed solvent (ethanol/ethylene glycol = 9/1, *v*/*v*). Thereafter, the film was annealed at 500 °C for 45 min. The inkjet-printed mp-SrTiO_3_/TiO_2_ ETL was prepared based on the same ink preparation and printing procedure as for the SrTiO_3_ ETL, except that a small amount of TiO_2_ was added into the SrTiO_3_-based inks. For SnO_2_-based devices, a single SnO_2_ ETL was used instead of the bilayer ETLs. The single layer was inkjet-printed using inks of diluted 15% tin oxide colloidal dispersion in a mixture of deionized water and ethylene glycol (9/1, *v*/*v*). A small amount of Triton X100 was added to adjust the surface tension of the SnO_2_ inks. Afterwards, the printed SnO_2_ film was annealed in a furnace at 220 °C for 1 h.

For reference TiO_2_-based devices, the fabrication procedure of the bilayer TiO_2_ ETLs in the literature [51] was followed. The compact TiO_2_ layer was deposited by spray pyrolysis at 450 °C with a solution of 0.2 M titanium (IV) isopropoxide and 2 M acetylacetone in isopropyl alcohol. Thereafter, the diluted TiO_2_ nanoparticle solution (Dyesol DSL 30NR-T, TiO_2_ paste/absolute ethanol = 1/5.5) was spin-coated at 4500 rpm for 30 s. The substrate was immediately transferred to a hotplate at 80 °C for 15 min and annealed at 500 °C for 30 min.

For the preparation of the perovskite precursors and the deposition of the perovskite layers, we followed the procedure described in the literature [51]. The perovskite precursor was prepared by dissolving 1.1 M PbI_2_, 1 M FAI, 0.2 M PbBr_2_ and 0.2 M MABr in a mixed solvent (N,N-dimethylformamide/dimethyl sulfoxide = 4/1, *v*/*v*). Seventy-five μL of the perovskite precursor was spread onto the substrate and spin-coated at 4500 rpm for 30 s, and subsequently 125 μL of chlorobenzene was sprayed onto the perovskite film during 15 s. The resulting perovskite film was immediately dried on a hotplate at 100 °C for 30 min. A hole transport layer was spin-coated at 4000 rpm for 30 s using a solution consisting of 70 mM Spiro-OMeTAD, 20 mM LiTFSI, 200 mM TBP, and 2 mM FK 209 Co(III) TFSI in chlorobenzene. An 80 nm Au electrode was deposited under vacuum by thermal evaporation (Edwards Auto 306). The final devices are displayed in Figure S1.

### 2.4. Characterization

XRD traces of the TiO_2_ compact layers were recorded by a grazing incidence X-ray diffractometer (Siemens D5000, Siemens, Munich, Germany) employing Cu Kα radiation (λ = 1.5406 Å). The morphology of the printed films, the cross-section images and the thickness of devices were studied by a combined focused ion beam/scanning electron microscope (FIB/SEM, FEI Nova 600 Nanolab, FEI Company, Eindhoven, The Netherlands). The working area of the solar cells was defined by a mask of 0.126 cm^2^ and the active area was illuminated under an AM 1.5G solar simulator (Newport 91160-1000) with an incident light density of 100 mW/cm^2^. Photocurrent density-voltage (*J-V*) characteristics were collected by a Keithley 2400 source-measure unit.

## 3. Results and Discussion

**Figure 1:**
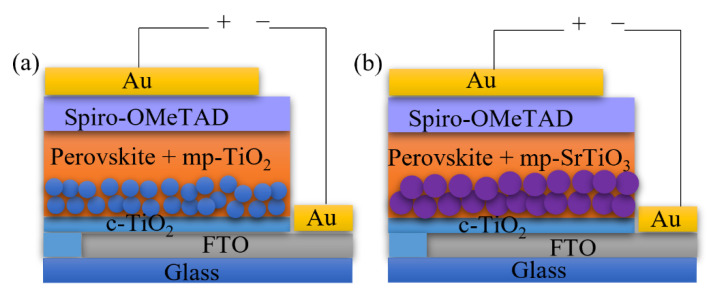
Architecture of (**a**) mp-TiO_2_- and (**b**) mp-SrTiO_3_-based PSC devices.

Bilayer TiO_2_ electron transport layers improve the device performance according to previous studies [3,4]. In this work, we applied the bilayer structure for different ETL materials consisting of a compact blocking layer and a mesoporous scaffold layer, as shown in Figure 1. Although considerable attempts were made to print the mesoporous TiO_2_ ETL for perovskite solar cells and dye-sensitized solar cells [38,52,53,54,55], less attention was paid to inkjet printing of other ETL materials, such as SrTiO_3_ and SnO_2_. In this work, we deposited the bilayer TiO_2_ ETLs by inkjet printing and optimized the printing processes by monitoring the photovoltaic performance of the inkjet-printed devices, and thereafter applied the optimized printing procedure for inkjet printing of mp-SrTiO_3_ and SnO_2_ ETLs.

**Figure 2:**
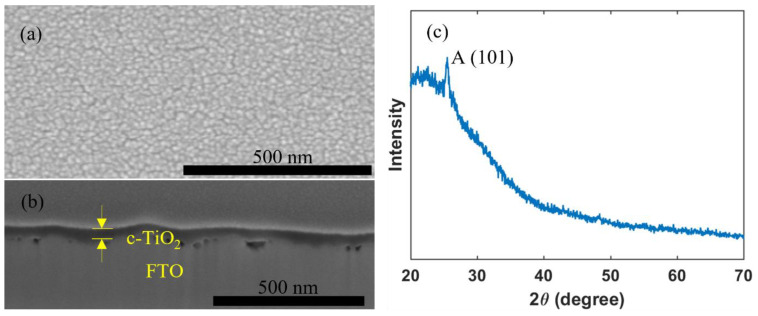
(**a**) Top view; (**b**) cross section SEM images; and (**c**) XRD pattern of an inkjet-printed c-TiO_2_ layer.

A c-TiO_2_ layer may effectively work as a hole blocking layer, which helps to reduce recombination losses and thus to improve photovoltaic performance, and thus commonly used in high performance PSCs [3,4,56,57]. Figure 2 shows that a uniform and pinhole-free c-TiO_2_ layer with a thickness of 50 nm can be successfully deposited by inkjet printing, and that the compact TiO_2_ layer crystallized with an anatase crystal structure.

**Figure 3:**
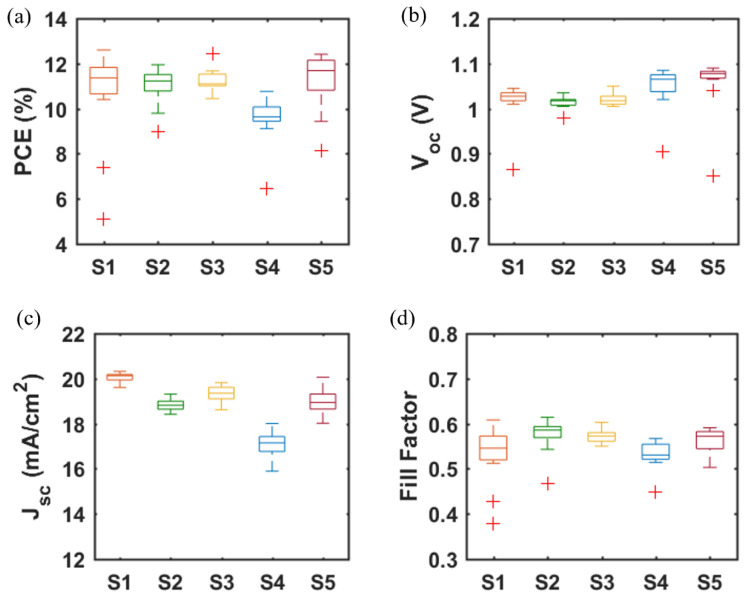
(**a**) PCE, (**b**) *V_OC_*, (**c**) *J_SC_*, and (**d**) *FF* of PSCs based on mp-TiO_2_ and mp-SrTiO_3_ ETLs inkjet-printed from nanoparticle inks with IPE or EtOH:EG as solvents. Data are obtained from 12 devices for each type of PSC. (Boxplots, red + signs represent outliers).

**Figure 4:**
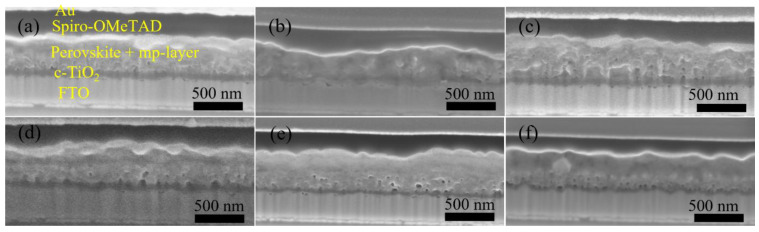
Cross-section SEM images of Device (**a**) S1, (**b**) S2, (**c**) S3, (**d**) S4, (**e**) S5, and (**f**) a spin-coated, TiO_2_-based PSC.

A major challenge for inkjet printing of high-quality thin films is to eliminate the ‘coffee-ring’ effect, which often occurs when the solvent of a drop containing dispersed solids evaporates and the nonvolatile solid components assemble at the periphery of the drop due to the outward capillary flow and pinned contact line [58,59]. To suppress the coffee-ring effect and control the film morphology, intense efforts were made in the literature and different approaches were proposed, such as solvent composition engineering [60,61], contact line depinning [59,62], evaporation temperature adjustment [63,64], and particle modification [65]. For inkjet printing, the optimization of the ink properties is the most common strategy to improve the film uniformity. Therefore, the selection of solvents for the solutions, and especially for nanoparticle dispersions, is crucial. The emphasis was focused on the physicochemical properties of the solvents, such as the viscosity and surface tension [66,67], which control the ink printability, film formation, and clogging mitigation of the nozzles within the printhead. A cosolvent system is widely used to control the morphology because of the inward Marangoni flow induced by the surface-tension gradients [53,54]. In the present work, we optimized the solvent composition for nanoparticle suspensions aimed at high device performance. The viscosity and surface tension of the inks were in the range of 1–25 mPa·s and 20–50 mN·m^−1^. Table 1 and Figure 3 demonstrate the effect of the solvent 2-isopropoxyethanol (IPE) and the mixed solvent ethanol: ethylene glycol (EtOH:EG = 9:1, *v*:*v*), used for dispersing TiO_2_ nanoparticles, on the photovoltaic performance of TiO_2_-based devices. For each set of solvents, 12 devices were manufactured. The average and champion photovoltaic parameters, including *V_OC_*, *J_SC_*, fill factor (*FF*) and PCE, are listed in Table 1 and the statistical distribution is illustrated in Figure 3. The series resistance (*R_s_*) and shunt resistance (*R_sh_*) are also estimated from the inverse of the slope of the *J-V* curves at the region of the *Voc* and the *Jsc*, respectively. Although Device S1 displayed a higher champion PCE than Device S2 (Figure S2), Device S2 offered a slightly higher average PCE of 10.97%, as compared to 10.64% of Device S1. From Figure 4a,b, the difference in the drying mechanics of the solvent IPE and EtOH:EG leads to also a difference in the microstructure (Figure S3) of the resulting mp-TiO_2_ film. A thicker ETL could allow more perovskite to be absorbed to absorb more incident light but may also lead to a larger series resistance causing Device S1 to exhibit a higher *J_SC_* and a lower *FF* than Device S2. With a higher TiO_2_ nanoparticle ink concentration, Device S3 showed an increase in *J_SC_* and a decrease in *FF* due to the formation of a thicker mesoporous TiO_2_ layer, as compared to Device S2. Eventually, as seen in Figure 3, although there is no considerable difference in PCE between Device S1, S2, and S3, Device S3 showed the best reproducibility ascribed to the microstructure of mp-TiO_2_(Figure 4c) inkjet-printed with TiO_2_ inks based on the mixed solvent EtOH:EG.

Furthermore, we fabricated SrTiO_3_-based Device S4 and Device S5 by inkjet printing of SrTiO_3_ nanoparticle inks with IPE and EtOH:EG as solvents, respectively. From Figure 3, it can be noted that Device S5 exhibits a higher efficiency with substantial improvements in all photovoltaic parameters, as compared to that of Device S4. A capping layer of perovskite on top of the ETL is beneficial for the improved performance because direct contact between ETL and the hole transport layer thereby is avoided, and thus recombination losses reduced. As seen in Figure 4d,e, the more porous structure of the mp-SrTiO_3_ films in Device S4 (Figure S3) results in a thinner capping perovskite layer, thereby causing more pronounced recombination losses and consequently accounting for the lower device performance.

**Figure 5:**
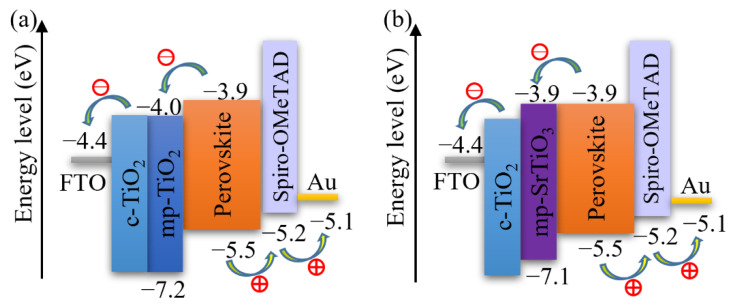
Schematics of the energy levels of (**a**) mp-TiO_2_- and (**b**) mp-SrTiO_3_-based PSCs.

Figure 3 also demonstrates that the photovoltaic performance of the SrTiO_3_-based Device S5 is comparable to that of TiO_2_-based devices. The *V_OC_* of SrTiO_3_-based solar cells is higher than that of the TiO_2_-based counterparts. A reason for the higher photovoltages is that a higher conduction band edge energy level and a smaller band edge offset of the SrTiO_3_ (Figure 5) will result in a higher *V_OC_* [17,18]. Another reason is that a capping perovskite layer is clearly observed in SrTiO_3_-based devices (Figure 4d,e) but not in TiO_2_-based devices (Figure 4b,c), thereby reducing the direct shunt loss path and yielding a higher *V_OC_*. The lower *J_SC_* obtained from SrTiO_3_-based devices as compared to that of TiO_2_-based devices is because of the limited loading of perovskite, caused by the smaller effective surface area of mp-SrTiO_3_ owing to the larger SrTiO_3_ nanoparticles [17,68].

**Figure 6:**
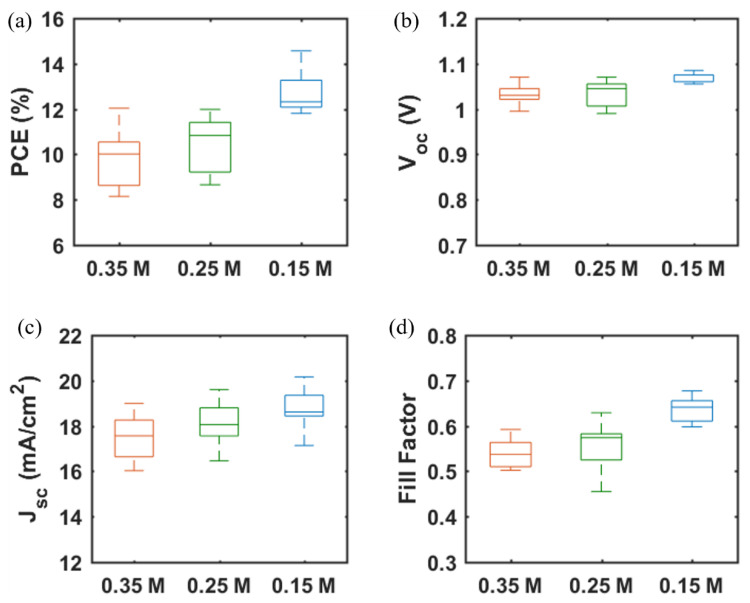
(**a**) PCE, (**b**) *V_OC_*, (**c**) *J_SC_*, and (**d**) *FF* of PSCs based on mp-SrTiO_3_ ETLs inkjet-printed with SrTiO_3_ nanoparticle inks of different concentrations. Data are obtained from 9 devices of each type.

**Figure 7:**
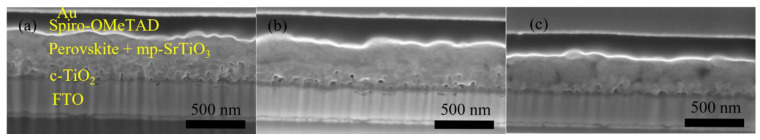
Cross-section SEM images of SrTiO_3_-based PSCs inkjet-printed using (**a**) 0.35 M, (**b**) 0.25 M, and (**c**) 0.15 M inks.

**Figure 8:**
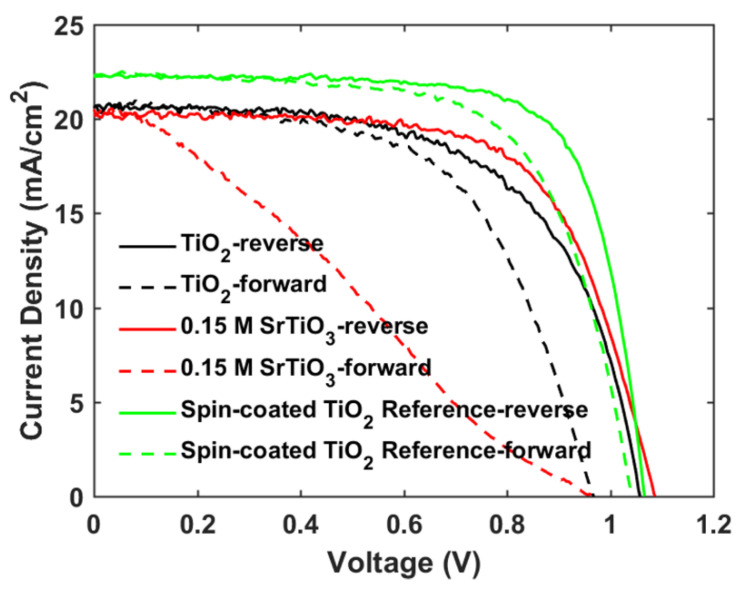
Reverse and forward scan *J-V* curves of mp-TiO_2_- and mp-SrTiO_3_-based PSCs.

The photovoltaic performance of mp-SrTiO_3_-based PSCs was further optimized. As seen in Table 2 and Figure 6, with lower concentrations of SrTiO_3_ in the nanoparticle inks, the performance of mp-SrTiO_3_-based PSCs is improved with higher *V_OC_*, *J_SC_* and *FF*. The main reason can be attributed to the ink concentrations which influence the thickness and microstructure of the inkjet-printed mp-SrTiO_3_ layer (Figure 7). Furthermore, inkjet-printed SrTiO_3_-based devices based on a 0.15 M SrTiO_3_ ink showed a PCE of 14.56%. Higher *V_OC_* is obtained for PSCs manufactured from 0.15 M SrTiO_3_ inks as compared to that of TiO_2_-based devices, as mentioned before. However, the recorded *J_SC_* is much lower since SrTiO_3_ nanoparticles with a large size express a smaller effective surface area, which limits the overall interfacial area between the perovskite layer and the ETL [20]. Therefore, this can result in an inefficient charge transfer in SrTiO_3_-based devices accounting for the pronounced scan-direction hysteresis observed, as shown in Figure 8, and the large difference in the overall performance for the two opposite scan directions. This dependence on the scan direction is much more pronounced in devices made from 0.15 M SrTiO_3_ inks than in TiO_2_-based ones. Another reason could be that SrTiO_3-_based cells demonstrate a larger *R_s_* than TiO_2_-based ones. A higher *R_s_* causes larger charge loss at high bias voltages and results in an inefficient charge transfer in the forward scan direction. In addition, the resulting SrTiO_3_ ETL (Figure 7c) printed from 0.15 M SrTiO_3_ inks is quite thin at some positions, which may cause the direct contact between the compact layer and the perovskite layer and then lead to recombination losses, thereby resulting in the pronounced hysteresis in SrTiO_3_-based PSCs.

**Figure 9:**
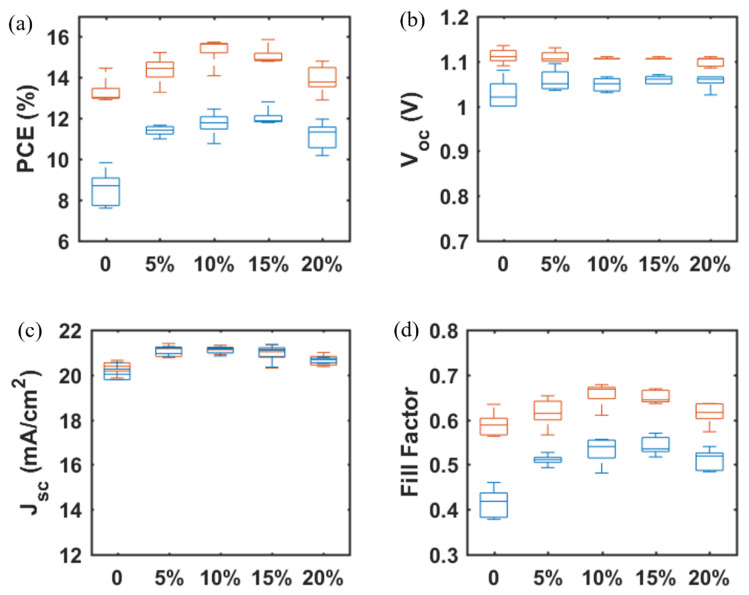
TiO_2_ concentration-dependent photovoltaic parameters (**a**) PCE, (**b**) *V_OC_*, (**c**) *J_SC_*, and (**d**) *FF* for mp-SrTiO_3_/TiO_2_-based PSCs. Red boxes and blue boxes represent data from reverse and forward scan directions, respectively.

To increase the *J_SC_* and suppress the hysteresis observed in SrTiO_3_-based PSCs, a small amount of TiO_2_ nanoparticles was added to the SrTiO_3_ nanoparticle inks to generate a mixed mesoporous SrTiO_3_/TiO_2_ ETL [21]. From Table 3 and Figure 9, the photovoltaic performance of SrTiO_3_/TiO_2_-based PSCs is improved as compared to SrTiO_3_-based PSCs, and the devices display a highest PCE of 15.73% with *V_OC_* = 1.11V, *J_SC_* = 20.99 mA/cm^2^ and *FF* = 0.68 (TiO_2_ concentration is 10 wt.% with respect to SrTiO_3_). The *V_OC_* shows a negligible change, while both *J_SC_* and *FF* increase and then decrease with TiO_2_ concentration ranging from 0 to 20%. Firstly, with introducing the smaller TiO_2_ nanoparticles to the SrTiO_3_ ETL, the interfacial area between the perovskite layer and the ETL will increase, and thus the *J_SC_* is expected to increase. The smaller TiO_2_ nanoparticles could also fill the pores between the larger SrTiO_3_ nanoparticles and as well at the surface of the mesoporous ETL, which is beneficial for increasing the *FF*. With the TiO_2_ concentration further increased from 10% to 20%, the electrical resistance of the SrTiO_3_/TiO_2_ ETL will increase, resulting in a decrease in *J_SC_* and *FF*.

**Figure 10:**
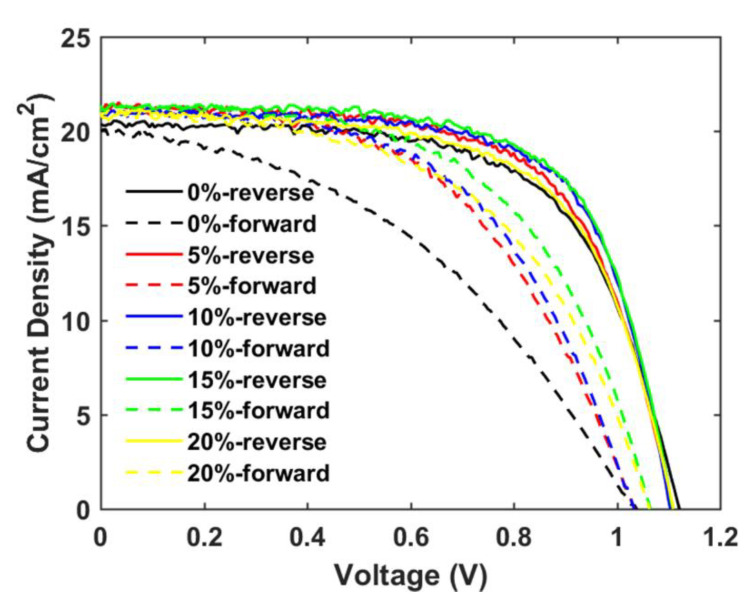
Reverse and forward scan *J-V* curves of champion cells of mp-SrTiO_3_/TiO_2_-based PSCs inkjet-printed with inks of different TiO_2_ concentrations.

The main objective of the formulating nanoparticle mixtures, the severe hysteresis problem, is indeed found to become suppressed for the mixed SrTiO_3_/TiO_2_-based PSCs, as shown in Table 4 and Figure 10. A hysteresis index is normally used to describe the performance difference between the reverse and forward scan direction and is defined by

(1) $$\mathrm{HI}=\frac{PCE\left|reverse-PCE\right|forward}{PCE|reverse}$$

where PCE|reverse and PCE|forward represent the power conversion efficiency from the reverse and forward scan directions, respectively. The hysteresis index decreases by 34% when the TiO_2_ concentration is increased from 0% to 10%, and the charge-transfer ability of the mp-SrTiO_3_/TiO_2_ ETL apparently is improved.

**Figure 11:**
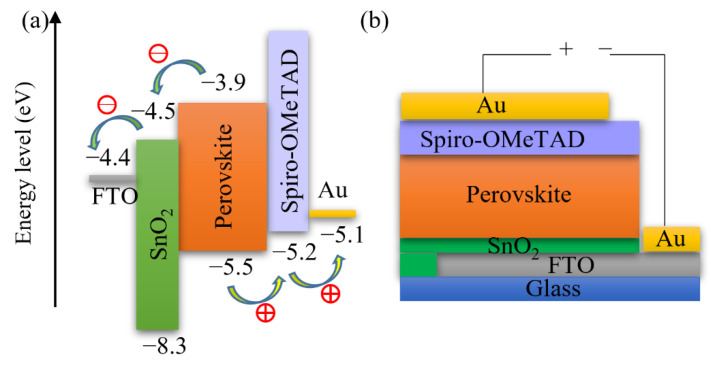
Schematics of (**a**) energy levels and (**b**) configuration of SnO_2_-based PSCs.

**Figure 12:**
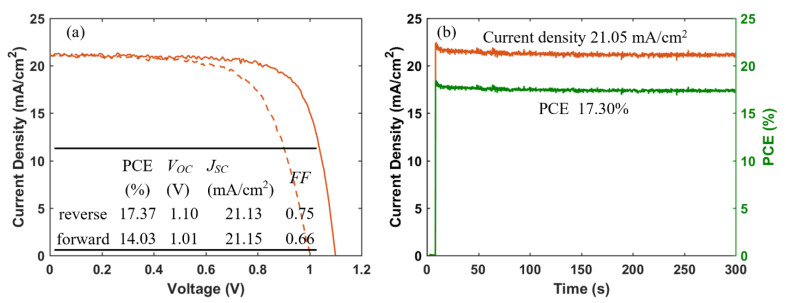
(**a**) *J-V* curves (solid and dashed lines represent data from reverse and forward scan, respectively), and (**b**) steady-state current density (at a bias of 0.925 V) and PCE of champion cell of PSCs based on inkjet-printed SnO_2_ ETLs.

**Figure 13:**
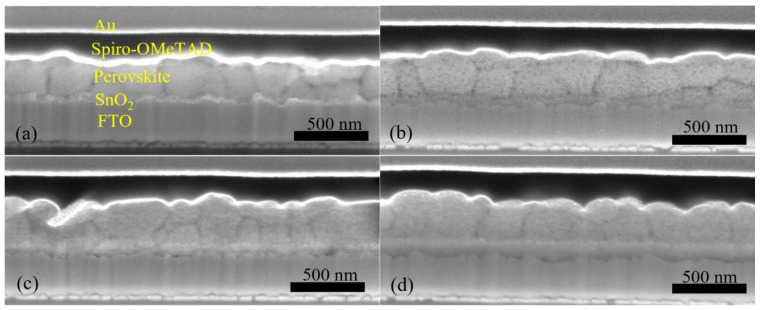
Cross-sectional SEM images for PSCs based on inkjet-printed SnO_2_ ETLs prepared from (**a**) 0.375%, (**b**) 0.75%, (**c**) 1.5%, and (**d**) 3% precursor inks.

Furthermore, we fabricated PSCs based on an inkjet-printed SnO_2_ ETL layer with a configuration as illustrated in Figure 11. Also in this system, we used a cosolvent system for printing the SnO_2_ thin films from the commercial SnO_2_ colloidal dispersion. Water works as the main solvent, and ethylene glycol and Triton are used to control the drying properties and to adjust the viscosity and surface tension for high-quality printing results. The details for preparing the SnO_2_ ink are described in the experimental section. The top SEM image of the inkjet-printed SnO_2_ thin film is shown in Figure S4. By optimizing the SnO_2_ thickness through changes of the ink concentration, we obtained a best performing cell with a PCE of 17.37% with a *V_OC_* of 1.10V, *J_SC_* of 21.13 mA/cm^2^, and *FF* of 0.75, and the *J-V* characteristics are shown in Figure 12a. We also recorded the steady-state current density of the champion cell at the maximum power point *V_mpp_* = 0.925 V. As seen in Figure 12b, a steady-state current density of 21.05 mA/cm^2^ and a stabilized PCE of 17.30% were obtained, which agree well with the *J_SC_* and PCE extracted from the *J-V* experiments. The average photovoltaic parameters of PSCs based on SnO_2_ ETLs printed with inks of different concentrations are shown in Table 5. When the SnO_2_ ink concentration is increased from 0.375% to 3%, the PCE (Figure S5) of SnO_2_-based PSCs decreases continuously. As seen in the cross-sectional SEM images in Figure 13, the SnO_2_ thickness increases from 30 nm to 130 nm when the ink concentration is increased from 0.375% to 3%. Too thick SnO_2_ films result in large sheet resistances, hampering charge transfer, thereby leading to lower PCEs.

As compared to that of TiO_2_- and SrTiO_3_-based PSCs, the SnO_2_-based congeners show less hysteresis, as well as better performance because of the superior electrical properties of the SnO_2_ material, such as high electron mobility [26]. Therefore, highly efficient perovskite solar cells based on inkjet-printed SnO_2_ ETLs should be attainable after a thorough optimization.

## 4. Conclusions

In this work, we developed the inkjet printing processes for TiO_2_, SrTiO_3_, and SnO_2_ ETLs. By optimizing the PSC performance based on the printed ETLs, the drying properties of cosolvent inks are beneficial for uniform film formation of the mesoporous TiO_2_ and SrTiO_3_ ETLs. Although SrTiO_3_-based PSCs perform better than TiO_2_-based ones, the former devices suffer from low current density and severe scan-direction hysteresis. PSCs based on the mixed mesoporous SrTiO_3_/TiO_2_ ETL can mitigate these problems and offer a higher PCE than the SrTiO_3_-only based devices. Furthermore, the strategy using cosolvent inks was also applied for inkjet printing of SnO_2_ ETLs. The best performing SnO_2_-based PSCs displayed an optimal PCE of 17.37% and low scan-direction hysteresis. In summary, the printing processes for different ETL materials were developed and will be used for fully inkjet-printed PSCs in our future work.

## Figures and Tables

**Table 1:** Photovoltaic parameters from the current density-voltage (*J-V*) characteristics of mp-TiO_2_- and mp-SrTiO_3_-based PSCs. Each average value is based on 12 devices.

	Ink Concentration	Solvent		PCE (%)	*V_OC_* (V)	*J_SC_* (mA/cm^2^)	*FF*	*R_s_* (Ω·cm^2^)	*R_sh_* (kΩ·cm^2^)
S1	0.25M TiO_2_	IPE	average	10.64 ± 2.21	0.99 ± 0.11	20.06 ± 0.20	0.53 ± 0.07		
			champion	12.60	1.04	19.83	0.61	7.8	2.0
S2	0.25M TiO_2_	EtOH:EG	average	10.97 ± 0.84	1.01 ± 0.01	18.83 ± 0.26	0.57 ± 0.04		
			champion	11.94	1.04	18.95	0.61	8.0	0.9
S3	0.35M TiO_2_	EtOH:EG	average	11.26 ± 0.49	1.02 ± 0.01	19.30 ± 0.36	0.57 ± 0.01		
			champion	12.42	1.05	19.63	0.60	7.7	2.0
S4	0.25M SrTiO_3_	IPE	average	9.54 ± 1.07	1.05 ± 0.05	17.08 ± 0.57	0.53 ± 0.03		
			champion	10.76	1.08	17.48	0.57	12	0.4
S5	0.25M SrTiO_3_	EtOH:EG	average	11.25 ± 1.30	1.06 ± 0.07	18.99 ± 0.63	0.56 ± 0.03		
			champion	12.41	1.08	20.06	0.57	16	1.0

**Table 2:** Photovoltaic parameters from *J-V* characteristics of mp-SrTiO_3_-based PSCs inkjet-printed with inks of different concentrations. Each average value is based on 9 devices.

Sample		PCE (%)	*V_OC_* (V)	*J_SC_* (mA/cm^2^)	*FF*	*R_s_* (Ω·cm^2^)	*R_sh_* (kΩ·cm^2^)
0.35M SrTiO_3_	average	9.81 ± 1.34	1.03 ± 0.02	17.51 ± 1.05	0.54 ± 0.03		
	champion	12.04	1.07	18.99	0.59	11	0.6
0.25M SrTiO_3_	average	10.33 ± 1.28	1.04 ± 0.03	18.06 ± 1.00	0.55 ± 0.05		
	champion	11.98	1.07	17.79	0.63	11	3.3
0.15M SrTiO_3_	average	12.75 ± 0.97	1.07 ± 0.01	18.77 ± 0.84	0.63 ± 0.03		
	champion	14.56	1.08	20.16	0.67	9.7	5.0

**Table 3:** Average photovoltaic parameters for mp-SrTiO_3_/TiO_2_-based PSCs inkjet-printed with inks of different TiO_2_ concentrations. Each average value is based on 5 devices.

Sample	Scan Direction	PCE (%)	*V_OC_* (V)	*J_SC_* (mA/cm^2^)	*FF*	*R_s_* (Ω·cm^2^)	*R_sh_* (kΩ·cm^2^)
SrTiO_3_/0% TiO_2_	reverse	13.31 ± 0.65	1.11 ± 0.02	20.33 ± 0.30	0.59 ± 0.03		
	forward	8.54 ± 0.90	1.03 ± 0.03	20.06 ± 0.32	0.41 ± 0.03		
	champion	14.46	1.12	20.38	0.63	9.7	2.0
SrTiO_3_/5% TiO_2_	reverse	14.37 ± 0.70	1.11 ± 0.01	21.00 ± 0.25	0.62 ± 0.03		
	forward	11.39 ± 0.26	1.06 ± 0.02	21.10 ± 0.19	0.51 ± 0.01		
	champion	15.23	1.10	21.09	0.65	8.3	1.4
SrTiO_3_/10% TiO_2_	reverse	15.34 ± 0.70	1.11 ± 0.00	21.11 ± 0.17	0.66 ± 0.03		
	forward	11.73 ± 0.61	1.05 ± 0.02	21.09 ± 0.16	0.53 ± 0.03		
	champion	15.73	1.10	20.99	0.68	7.0	2.5
SrTiO_3_/15% TiO_2_	reverse	15.07 ± 0.45	1.11 ± 0.00	20.94 ± 0.39	0.65 ± 0.01		
	forward	12.05 ± 0.42	1.06 ± 0.01	20.98 ± 0.38	0.54 ± 0.02		
	champion	15.86	1.11	21.36	0.67	7.3	3.3
SrTiO_3_/20% TiO_2_	reverse	13.93 ± 0.72	1.10 ± 0.01	20.62 ± 0.25	0.61 ± 0.03		
	forward	11.12 ± 0.69	1.06 ± 0.02	20.64 ± 0.12	0.51 ± 0.02		
	champion	14.80	1.11	20.99	0.64	8.7	1.0

**Table 4:** Average hysteresis index for mp-SrTiO_3_/TiO_2_-based PSCs inkjet-printed with inks of different TiO_2_ concentrations.

Sample	SrTiO_3_/0% TiO_2_	SrTiO_3_/5% TiO_2_	SrTiO_3_/10% TiO_2_	SrTiO_3_/15% TiO_2_	SrTiO_3_/20% TiO_2_
Hysteresis index	0.36	0.21	0.24	0.20	0.20

**Table 5:** Photovoltaic parameters for PSCs based on inkjet-printed SnO_2_ ETLs prepared from different ink concentrations. Averages are based on different number of cells, as indicated in second column.

Cells	Numbers of Devices	Scan Direction	PCE (%)	*V_OC_* (V)	*J_SC_* (mA/cm^2^)	*FF*	HysteresisIndex	*R_s_* (Ω·cm^2^)	*R_sh_* (kΩ·cm^2^)
0.375%	14	reverse	16.61 ± 0.70	1.10 ± 0.01	20.85 ± 0.26	0.73 ± 0.02	0.16 ± 0.03		
		forward	13.93 ± 0.66	1.02 ± 0.01	21.01 ± 0.24	0.65 ± 0.03			
		champion	17.37	1.10	21.13	0.75		4.9	5.0
0.75%	7	reverse	15.57 ± 0.66	1.06 ± 0.00	20.42 ± 0.36	0.72 ± 0.02	0.21 ± 0.02		
		forward	12.29 ± 0.45	0.94 ± 0.01	20.42 ± 0.45	0.64 ± 0.02			
		champion	16.53	1.06	20.96	0.74		5.7	2.5
1.5%	8	reverse	11.30 ± 0.77	0.95 ± 0.03	18.81 ± 0.46	0.63 ± 0.03	0.13 ± 0.04		
		forward	9.85 ± 0.48	0.85 ± 0.02	18.73 ± 0.56	0.62 ± 0.03			
		champion	12.59	0.99	19.58	0.65		9.5	1.1
3%	7	reverse	10.32 ± 0.81	0.91 ± 0.04	17.82 ± 0.45	0.64 ± 0.05	0.06 ± 0.03		
		forward	9.73 ± 0.63	0.85 ± 0.03	17.72 ± 0.52	0.65 ± 0.04			
		champion	11.70	0.94	18.25	0.68		8.5	2.0

## Data Availability

The data presented in this study are available on request from the corresponding author.

## Supplementary Materials

The following are available online at https://www.mdpi.com/article/10.3390/ma14247525/s1, Figure S1: Device geometry of perovskite solar cells, Figure S2: *J-V* curves of champion PSCs based on mp-TiO_2_ and mp-SrTiO_3_ ETLs inkjet-printed from nanoparticle inks with IPE or EtOH:EG as solvents, Figure S3: SEM images of mesoporous layers inkjet-printed from (a) TiO_2_ inks with the solvent IPE, (b) TiO_2_ inks with the solvent EtOH:EG, (c) SrTiO_3_ inks with the solvent IPE, and (d) SrTiO_3_ inks with the solvent EtOH:EG, Figure S4: SEM image of inkjet-printed SnO_2_ thin film, Figure S5: *J-V* curves of champion PSCs based on inkjet-printed SnO_2_ ETLs prepared from 0.375%, 0.75%, 1.5%, and 3% precursor inks, Table S1: Technical information of the printhead XJ126/50 and XJ126/80. These parameters are obtained from the Xaar126 data sheet.
