# Supplementary material for: Inkjet-Printed Electron Transport Layers for Perovskite Solar Cells
Source: Materials (Basel). 2021 Dec 8;14(24):7525. doi: 10.3390/ma14247525 (PMC8704523; doi:10.3390/ma14247525)
Supplement: Supplementary file 1 [file materials-14-07525-s001.zip › materials-1456410-supplementary.pdf]

materials MDPI

Supplementary Materials

Inkjet-Printed Electron Transport Layers for Perovskite Solar Cells

Dongli Lu [1], Wei Zhang [2], Lars Kloo [2] and Liubov Belova [1,*]

[1] Department of Materials Science and Engineering, KTH Royal Institute of Technology, SE-10044 Stockholm, Sweden; donglil@kth.se
[2] Department of Chemistry, Applied Physical Chemistry, KTH Royal Institute of Technology, SE-10044 Stockholm, Sweden; wzha@kth.se (W.Z.); larsa@kth.se (L.K.)
* Correspondence: lyuba@kth.se

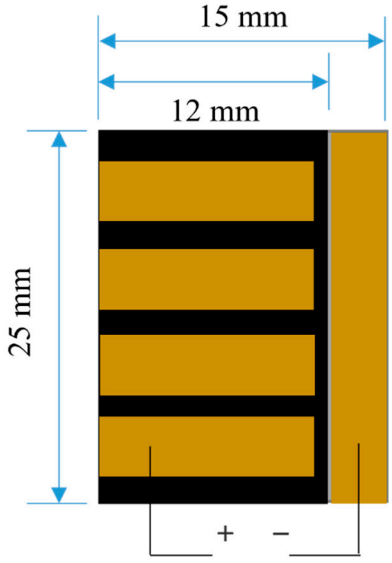

Figure S1. Device geometry of perovskite solar cells.

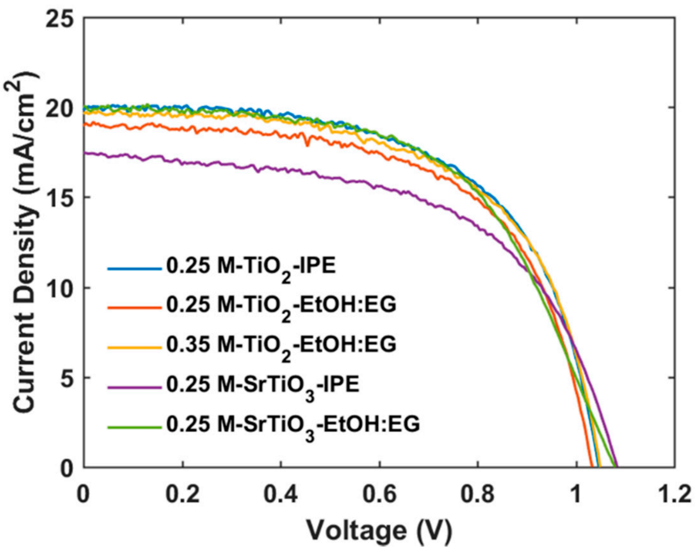

Figure S2. *J-V* curves of champion PSCs based on mp-TiO_2 and mp-$SrTiO_3$ ETLs inkjet printed from nanoparticle inks with IPE or EtOH:EG as solvents.

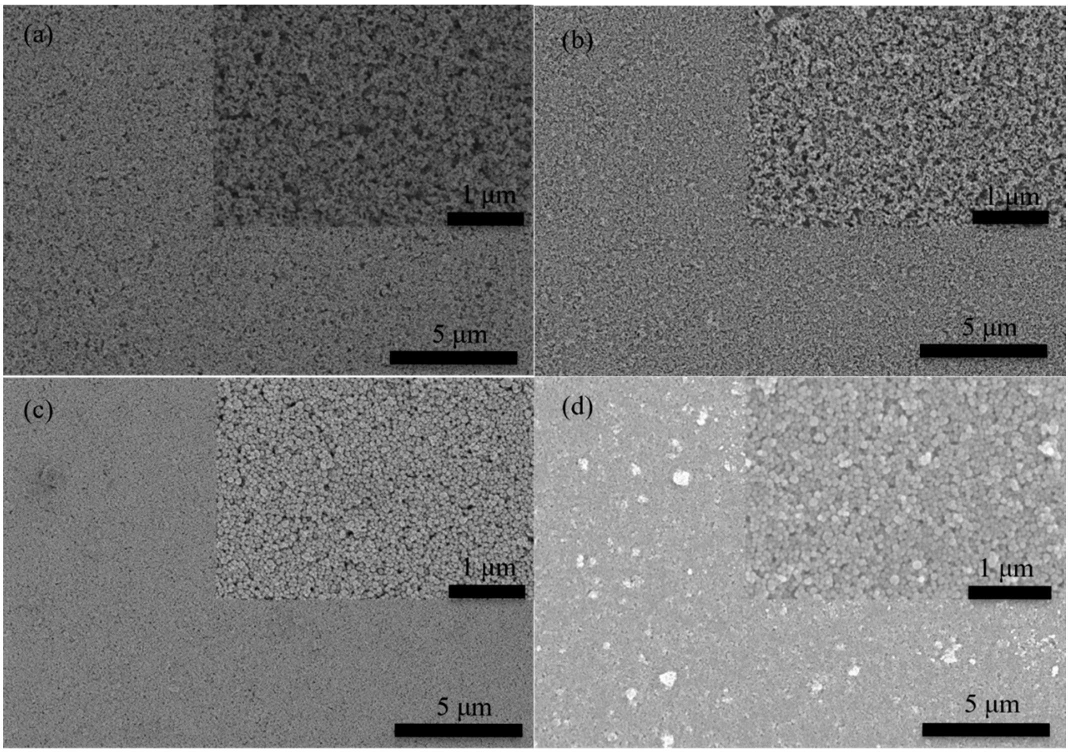

Figure S3. SEM images of mesoporous layers inkjet printed from (a) TiO_2 inks with the solvent IPE, (b) TiO_2 inks with the solvent EtOH:EG, (c) $SrTiO_3$ inks with the solvent IPE, and (d) $SrTiO_3$ inks with the solvent EtOH:EG.

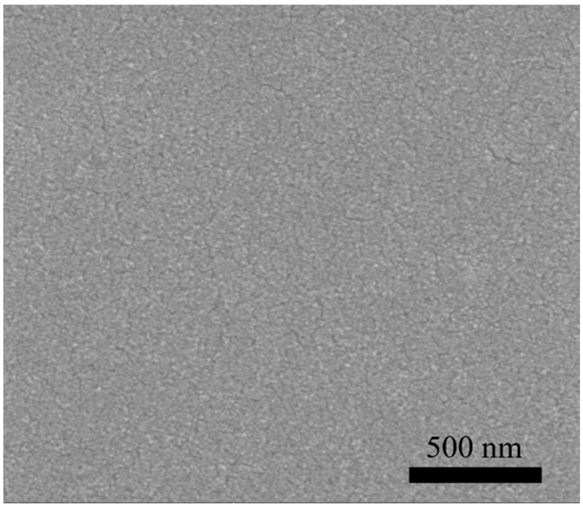

Figure S4. SEM image of inkjet-printed SnO_2 thin film.

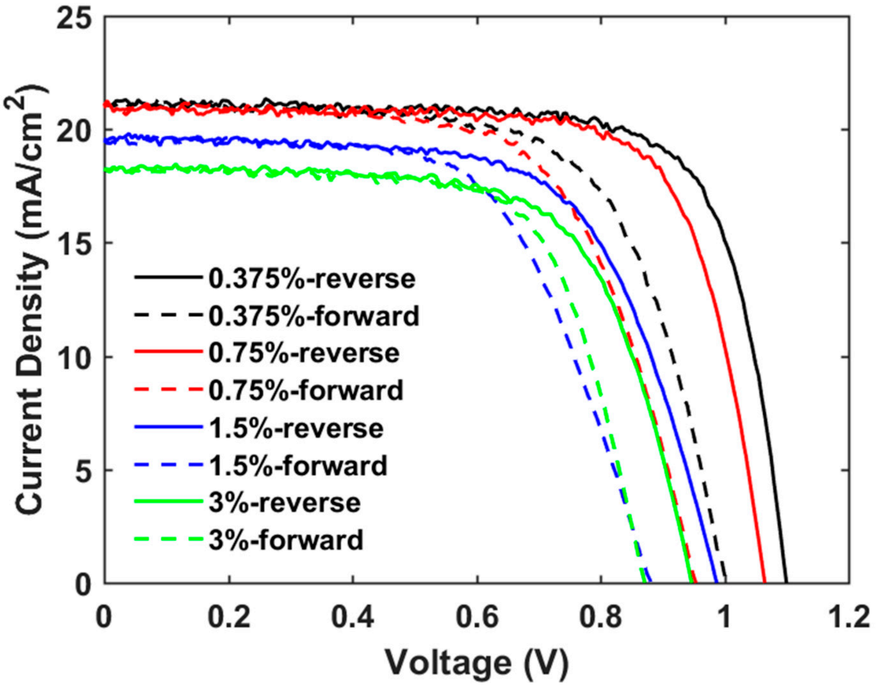

Figure S5. *J-V* curves of champion PSCs based on inkjet-printed SnO_2 ETLs prepared from 0.375%, 0.75%, 1.5%, and 3% precursor inks.

Table S1. Technical information of the printhead XJ126/50 and XJ126/80. These parameters are obtained from the Xaar126 data sheet.

	**XJ126/50**	**XJ126/80**
Active nozzles	126	126
Print swathe width (mm)	17.2	17.2
Nozzle pitch (µm)	137.3	137.3
Nozzle density (nozzles/inch)	185	185
Drop volume (pl)	50	80
Drop velocity (m/s)	6	6
